# Field-based and molecular evaluation of anthelmintic resistance in gastrointestinal strongyle nematodes of meat goats in Southern Thailand

**DOI:** 10.14202/vetworld.2025.2467-2478

**Published:** 2025-08-26

**Authors:** Narin Sontigun, Chalutwan Sansamur, Tunwadee Klong-Klaew, Raktham Mektrirat, Morakot Kaewthamasorn, Punpichaya Fungwithaya

**Affiliations:** 1 Office of Administrative Interdisciplinary Program on Agricultural Technology, School of Agricultural Technology, King Mongkut’s Institute of Technology Ladkrabang, Bangkok 10520, Thailand; 2 Akkhraratchakumari Veterinary College, Walailak University, Nakhon Si Thammarat 80160, Thailand; 3 Department of Parasitology and Entomology, Faculty of Public Health, Mahidol University, Bangkok 10400, Thailand; 4 Veterinary Academic Office, Faculty of Veterinary Medicine, Chiang Mai University, Chiang Mai 50100, Thailand; 5 Veterinary Research Center for Veterinary Biosciences and Veterinary Public Health, Faculty of Veterinary Medicine, Chiang Mai University, Chiang Mai 50100, Thailand; 6 Center of Excellence in Pharmaceutical Nanotechnology, Faculty of Pharmacy, Chiang Mai University, Chiang Mai 50200, Thailand; 7 Center of Excellence in Veterinary Parasitology, Department of Pathology, Faculty of Veterinary Science, Chulalongkorn University, Bangkok 10330, Thailand

**Keywords:** albendazole, allele-specific polymerase chain reaction, anthelmintic resistance, F200Y mutation, *Haemonchus contortus*, ivermectin, meat goats, Thailand, *Trichostrongylus colubriformis*, β-tubulin gene

## Abstract

**Background and Aim:**

Gastrointestinal nematodes (GINs) such as *Haemonchus contortus* and *Trichostrongylus colubriformis* are major health threats in goats, exacerbated by growing anthelmintic resistance (AR). Despite the widespread use of albendazole and ivermectin in Nakhon Si Thammarat, Southern Thailand, data on AR status in goats remain scarce. This study aimed to evaluate the resistance of GINs to albendazole and ivermectin using fecal egg count reduction (FECR) tests and to determine benzimidazole (BZ) resistance through molecular detection of the F200Y mutation in the β-tubulin isotype 1 gene.

**Materials and Methods:**

A total of 192 meat goats from six farms were randomly divided into four groups: untreated control, albendazole-treated, ivermectin-treated, and combination-treated. FECR was assessed on day 14 post-treatment. Larval cultures and semi-nested polymerase chain reaction (PCR) were used to identify nematode genera. BZ resistance was determined through allele-specific PCR on *H. contortus* and *T. colubriformis* third-stage larvae, pre- and post-albendazole treatment.

**Results:**

FECR revealed confirmed resistance (FECR < 95%) to albendazole (−35.48%–62.5%), ivermectin (−2.41%–51.47%), and their combination (−25%–48.36%) across all farms. *Haemonchus* and *Trichostrongylus* were the predominant genera post-treatment. Molecular analysis showed high pre-treatment frequencies of the F200Y resistance allele in *H. contortus* (75.0%–80.6%) and *T. colubriformis* (88.6%–100%), which reached 100% post-treatment. Susceptible genotypes were entirely eliminated following albendazole treatment.

**Conclusion:**

This is the first comprehensive study confirming widespread AR to albendazole and ivermectin in meat goats in southern Thailand. The fixation of the BZ resistance allele in both nematode species highlights the urgency for revising current deworming practices. Immediate adoption of integrated parasite management strategies, including drug rotation, targeted selective treatment, and exploration of alternative anthelmintics, is critical to mitigate economic losses and protect public health.

## INTRODUCTION

Gastrointestinal nematodes (GINs) represent a significant health challenge for goats globally, including in Thailand [1–5]. To control GIN infections, farmers primarily rely on three major classes of anthelmintics: benzimidazoles (BZs, e.g., albendazole), imidazothiazoles (e.g., levamisole), and macrocyclic lactones (MLs, e.g., ivermectin) [6]. However, anthelmintic resistance (AR) to all these drug classes – especially multidrug resistance (MDR) – has emerged as a critical concern in the goat industry worldwide, with rising reports from various countries [6–9].

Among GINs, strongyle nematodes, including *Cooperia* spp., *Haemonchus* spp., *Oesophagostomum* spp., and *Trichostrongylus* spp., are the most prevalent in goats [1, 2, 10, 11]. Notably, *Haemonchus contortus* and *Trichostrongylus colubriformis* are not only the most pathogenic but are also commonly associated with AR [7, 9]. These species are of additional concern due to their zoonotic potential, having been implicated in human trichostrongylosis cases [12–14].

The fecal egg count reduction (FECR) test remains the standard phenotypic method for evaluating AR across all anthelmintic classes [15, 16]. However, its limitations, including low sensitivity, high cost, and labor-intensive procedures, have spurred the development of molecular alternatives [7, 17]. For detecting benzimidazole (BZ) resistance, allele-specific polymerase chain reaction (AS-PCR) is widely employed to identify single-nucleotide polymorphisms (SNPs) in the β-tubulin isotype 1 gene, particularly at codons 200 (F200Y: TTC→TAC), 167 (F167Y: TTC→TAC), and 198 (E198A: GAA→GCA; E198L: GAG→TTG). Among these, the F200Y mutation is the most frequently reported and is considered a key genetic marker for BZ resistance in many countries [18–20]. Genetic studies on BZ resistance have largely focused on *H. contortus*, *Teladorsagia circumcincta*, and *T. colubriformis*, which dominate in both tropical and temperate climates [20, 21]. In contrast, reliable molecular markers for detecting resistance to MLs and imidazothiazoles remain underdeveloped.

Despite growing international awareness of AR in GINs, particularly in small ruminants, regional surveillance remains inconsistent, especially in Southeast Asia. In Thailand, albendazole and ivermectin have been the mainstay treatments for decades, promoted by the Department of Livestock Development for their broad-spectrum efficacy and affordability. However, repeated, prolonged, and often unsupervised use has led to increasing reports of reduced efficacy and AR in several provinces [22–25]. Southern Thailand, specifically Nakhon Si Thammarat, is a region with a high density of smallholder goat farms where anthelmintic use is widespread. Yet, there is a surprising lack of comprehensive, field-based AR assessments in this region.

Current knowledge is particularly limited regarding the molecular mechanisms underpinning BZ resistance in GIN populations in Southern Thailand. Most available studies have focused on clinical or parasitological outcomes without integrating molecular confirmation of resistant alleles. Only one previous investigation by Pitaksakulrat *et al*. [26] in Thailand employed AS-PCR to detect the F200Y mutation in the β-tubulin isotype 1 gene of *H. contortus* from goats, and no study has concurrently evaluated both *H. contortus* and *T. colubriformis* in a regional field context. Furthermore, no study has explored the combination treatment of albendazole and ivermectin or attempted to validate treatment outcomes with both FECR testing and molecular diagnostics. This lack of integrated, molecularly-informed AR surveillance limits the ability of veterinarians and policymakers to develop effective parasite control strategies and poses risks to animal productivity and public health.

This study was designed to address the critical lack of phenotypic and molecular data on AR in GINs of goats in southern Thailand. Specifically, it aimed to:

1. Evaluate the efficacy of albendazole, ivermectin, and their combination against GINs in meat goats in Nakhon Si Thammarat using the FECR test;
2. Identify the strongyle genera before and after treatment through larval culture, microscopy, and semi-nested PCR;
3. Assess BZ resistance by detecting the F200Y mutation in the β-tubulin isotype 1 gene using AS-PCR in *H. contortus* and *T. colubriformis* larvae.

The results of this study will contribute to the understanding of local AR patterns, support evidence-based treatment protocols, and promote the adoption of sustainable parasite control practices in Thai goat farming systems.

## MATERIALS AND METHODS

### Ethical approval

This study was approved by the Institutional Animal Care and Use Committee of Walailak University (Approval No. WU-ACUC-66056).

### Study period and location

**Figure 1:**
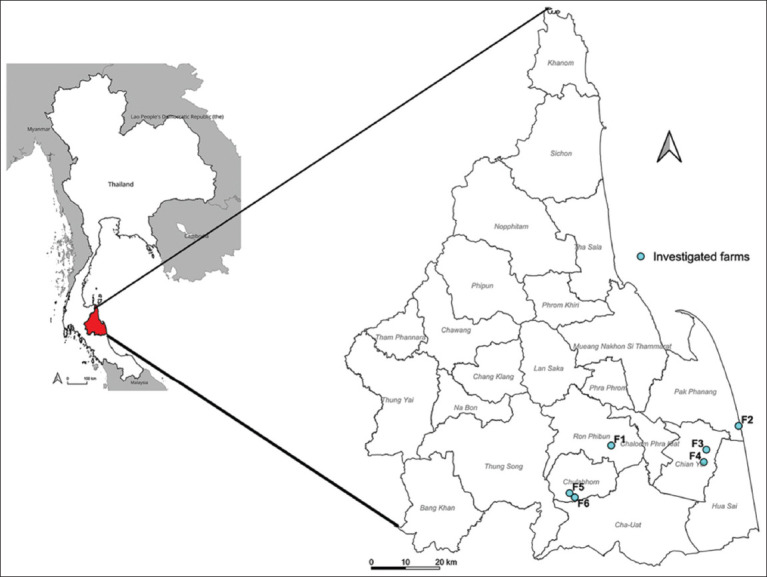
Map showing the locations of six meat goat farms in four districts of Nakhon Si Thammarat province, southern Thailand, including Ron Phibun (F1), Pak Phanang (F2), Chian Yai (F3 and F4), and Chulabhorn (F5 and F6) [Source: The map was generated using QGIS version 3.40.3 (https://qgis.org/), incorporating administrative boundary data for Thailand, neighboring countries, and Nakhon Si Thammarat Province, obtained from the Humanitarian Data Exchange (https://data.humdata.org)].

This study was conducted between April and August 2024 on six meat goat farms in four districts of Nakhon Si Thammarat province, Southern Thailand (Figure 1): Ron Phibun (1 farm; F1), Pak Phanang (1 farm; F2), Chian Yai (2 farms; F3 and F4), and Chulabhorn (2 farms; F5 and F6). Farm selection was based on unpublished data indicating a high prevalence of GI infections and routine anthelmintic use. Each farm housed between 50 and 100 goats. Four of the selected farms (F2, F3, F5, and F6) operated under a semi-intensive system, allowing goats to graze during the day (without pasture rotation) and housing them indoors at night. The remaining two farms (F1 and F4) followed an intensive system, keeping goats permanently in pens and supplying fresh grass as feed. All farmers independently purchased and administered anthelmintics, typically every 3–6 months (2–4 times/year). Each farm used albendazole and ivermectin consistently every year since herd establishment, ranging from 9 to 14 years. Anthelmintic dosages were estimated visually based on the weight of each goat, leading to frequent cases of underdosing or overdosing.

### Animal selection criteria

Meat goats (>6 months old) that had not been treated with anthelmintics in the past 8 weeks and had fecal egg counts (FEC) of >150 eggs per gram (EPG) were included in the study.

### Calculation of sample size

The sample size for assessing AR in GINs was calculated using G*Power software version 3.1.9.7 [27]. The calculation was based on a t-test with an alpha level of 0.05 and statistical power of 0.95. An effect size corresponding to an OR of 3.36 was used. The required sample size was 32 goats/farm.

### Design of anthelmintic treatment

On each farm, 32 goats were selected using simple random sampling and then randomly allocated into four groups of eight: untreated control, albendazole-treated (5.625 mg/kg orally), ivermectin-treated (0.2 mg/kg subcutaneously), and combination-treated with both drugs at the same doses. The control group was used to account for natural fluctuations in FEC. All goats, regardless of the treatment group, were housed under identical conditions. Local anthelmintic brands were used in the treatment groups, with dosages based on body weight and standard farmer practices administered per manufacturer’s instructions. Before dosing, all goats were weighed and uniquely identified using ear tags.

### Collection and storage of fecal samples

Fecal samples were collected directly from the rectum using polyethylene gloves, placed in labeled ziplock bags, stored in iceboxes, and transported to the Parasitology Laboratory, Akkhraratchakumari Veterinary College, Walailak University. The samples were refrigerated at 4°C and examined within 24 h of sample collection.

### FECR test

The FECR test was conducted in accordance with the World Association for the Advancement of Veterinary Parasitology guidelines for evaluating anthelmintic efficacy in ruminants [15, 28]. Approximately 10 g of feces was collected from each goat on days 0 (pre-treatment) and 14 (post-treatment). GIN egg counts were determined using the modified McMaster technique with a detection threshold of 50 EPG [29].

### Larval culture and morphological identification

Pooled fecal samples from each group at each farm were cultured before and after treatment to identify the presence of nematodes. Third-stage larvae (L3) were isolated from the cultures using the Baermann technique [29]. One hundred L3 were randomly selected from each pooled sample, examined under a light microscope (Nikon, USA), and identified at the genus level using the morphological key by van Wyk and Mayhew [30]. If fewer than 100 L3 were recovered, larval percentages were calculated based on the total available count. Larvae were exsheathed in 0.187% sodium hypochlorite for 3–5 min [31] and then rinsed 3 times with distilled water before molecular analysis.

### Genus confirmation using semi-nested PCR

To validate morphological identification, 2–3 representative L3s of *Haemonchus*, *Oesophagostomum*, and *Trichostrongylus* were selected from both the control and treatment groups (pre- and post-treatment) at each farm for molecular analysis. Genomic DNA was extracted using the SimpleWay gDNA Prep and PCR Set I Kit (Biofact, Daejeon, Republic of Korea) according to the manufacturer’s instructions. Larvae were lysed by adding 50 μL SLB buffer, 5 μL Proteinase K, and stainless-steel beads to each 2 mL tube, followed by homogenization in a TissueLyser LT (Qiagen, Hilden, Germany) at 50 Hz for 5 min. The samples were incubated at 99°C for 10 min, cooled for 2 min, and centrifuged at 12,879× *g* for 1 min. The supernatant was transferred to a clean tube and stored at −20°C until further analysis.

Semi-nested PCR was conducted to identify strongyle nematode genera using primers and protocols from Income *et al*. [2] (Table 1). Each PCR reaction consisted of 6.25 μL PCR master mix III (2×) (Biofact), 1–2 μL DNA template, 0.2 μM of Strongyle F2 and R3 primers (Table 1), and nuclease-free water to a final volume of 12.5 μL. Thermal cycling conditions included initial denaturation at 95°C for 2 min, followed by 35 cycles of 95°C for 20 s, 50°C for 30 s, and 72°C for 30 s, with a final extension at 72°C for 5 min. PCR products were resolved on 1.5% agarose gel in 1× TAE buffer, stained with SERVA DNA Stain G (Serva, Heidelberg, Germany), and visualized under ultraviolet (UV) light using the ChemiDoc Imaging System (Bio-Rad, Hercules, CA, USA). The reverse primer was replaced with a genus-specific primer (Table 1) in the semi-nested PCR, and 1 μL of diluted primary PCR product (1:50 in nuclease-free water) was used as the template under identical thermal cycling conditions.

**Table 1:** Strongyle detection primers by semi-nested PCR.

Genus	Primer name	Region	Sequence (5’–3’)	Size of the PCR product (bp)
All strongyles	Strongyle F2	5.8S rRNA	TGGTGAAATTTTGAACGCATAG	324–349
	Strongyle R3	28S rRNA	ATGCTTAAGTTCAGCGGGTA	
*Cooperia*	Cooper R	ITS2	CGAATACTACTATCTCCAACATG	293
*Haemonchus*	Haemo R	ITS2	GTACACTCAAATAGWGGCAACAT	227
*Oesophagostomum*	Oeso R	ITS2	CTCATCTAGAACGAGGATCACA	143
*Trichostrongylus*	Tricho R	ITS2	CAATATTTGAYAATGACCATTCG	128

ITS=Internal transcribed spacer, rRNA=Ribosomal RNA, PCR=Polymerase chain reaction, F=Forward primer, R=Reverse primer

### Detection of BZ resistance using AS-PCR

Due to budgetary constraints, 50% of *H. contortus* and *T. colubriformis* L3 recovered from each farm, both pre- and post-albendazole treatment, were randomly selected for evaluation. AS-PCR was conducted to detect mutations at codon 200 of the β-tubulin isotype 1 gene associated with BZ resistance, using primers and protocols described by Silvestre and Humbert [21] (Table 2). Genomic DNA was extracted from individual L3s using the Phire Tissue Direct PCR Master Mix Kit (Thermo Fisher Scientific, Waltham, MA, USA) according to the manufacturer’s instructions. Each larva was homogenized in a 2 mL tube containing 15 μL dilution buffer, 0.5 μL DNARelease Additive, and stainless-steel beads using a TissueLyser LT (Qiagen) at 50 Hz for 5 min. Samples were incubated at 98°C for 2 min and centrifuged. The resulting supernatant was transferred to a new tube and stored at −20°C for later use. Each PCR reaction included 6.25 μL Phire Tissue Direct PCR Master Mix (2×) (Thermo Fisher Scientific), 1–2 μL DNA template, 0.4 μM of each primer (Table 2), and nuclease-free water to a total volume of 12.5 μL.

**Table 2:** AS-PCR primers for BZ resistance in L3.

Species	Primer name	Sequence (5’–3’)
*H. contortus*	NS-Ph1Fw	GGAACGATGGACTCCTTTCG
	NS-Ph2Rv	GATCAGCATTCAGCTGTCCA
	S-Ph4Rv	ATACAGAGCTTCGTTGTCAATACAGA
	R-Ph3Fw	CTGGTAGAGAACACCGATGAAACATA
*T. colubriformis*	NS-Pc1Fw	GGAACAATGGATTCCGTTCG
	NS-Pc2Rv	GGGAATCGGAGGCAAGTCGT
	S-Pc4Rv	ATACAGAGCTTCGTTATCGATGCAGA
	R-Pc3fw	CTGGTAGAGAATACCGATGAAACATA

BZ=Benzimidazole, L3=Third-stage larvae, AS-PCR=Allele-specific polymerase chain reaction, *H. contortus*=*Haemonchus contortus*, *T. colubriformis*=*Trichostrongylus colubriformis*, NS=Non-specific fragment (750 bp), S=Susceptible-specific fragment (550 bp), R=Resistant-specific fragment (250 bp), Fw=Forward primer, Rv=Reverse primer

Thermal cycling involved an initial denaturation at 98°C for 5 min, followed by 40 cycles of 98°C for 5 s, annealing at 55°C (*T. colubriformis*) or 60°C (*H. contortus*) for 5 s, 72°C for 20 s, and a final extension at 72°C for 1 min. PCR products were separated on a 1.5% agarose gel in 1× TAE buffer, stained with SERVA DNA Stain G (Serva, Germany), and visualized under UV light using the ChemiDoc Imaging System (Bio-Rad). Band sizes corresponding to susceptible, resistant, and non-specific fragments were approximately 550, 250, and 750 base pair (bp), respectively. Homozygous resistant (RR) genotypes were identified by the presence of either only the 250-bp band or both the 250- and 750-bp bands. Homozygous susceptible (SS) genotypes were confirmed by the presence of only the 550-bp band or both 550- and 750-bp bands. Heterozygous resistant (RS) genotypes were identified by the presence of both 250- and 550-bp bands, with or without the 750-bp non-specific band.

### Statistical analysis

The anthelmintic efficacy was evaluated by calculating the reduction in FEC. The FECR percentage (%FECR) was calculated using the formula: 100 × (1 – [T2/T1]), where T2 is the mean EPG on day 14 and T1 is the mean EPG on day 0 for the treated group [32]. AR was considered confirmed if the %FECR was below 95% and the lower 95% confidence limit was below 90% [15]. If only one of these criteria was satisfied, the resistance was classified as suspected. If both criteria were unmet, the population was considered susceptible. All data were recorded and analyzed using Microsoft Excel 2019 (Microsoft Corporation, Redmond, WA, USA).

## RESULTS

### FECR results

**Figure 2:**
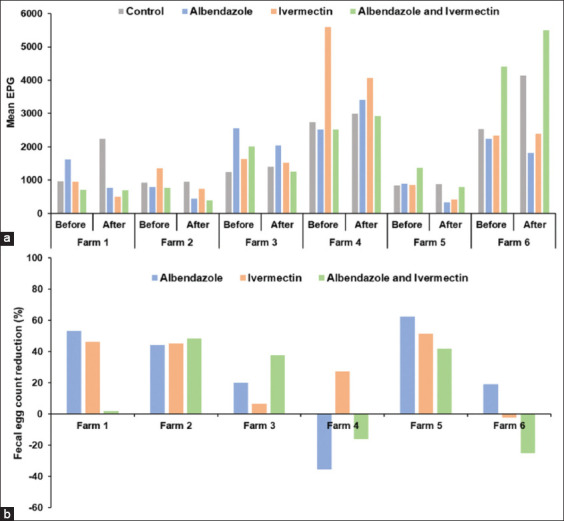
(a) Mean eggs per gram (EPG) counts before and after treatment and (b) fecal egg count reduction percentages for each anthelmintic agent across six meat goat farms.

All fecal samples (n = 192) contained strongyle-type eggs (100%), followed by *Eimeria* spp. (81.8%), *Strongyloides papillosus* (19.3%), and *Trichuris* spp. (5.2%). The FECR results for each anthelmintic treatment across the six meat goat farms are summarized in Table 3. All six farms showed FECR < 95% for all treatments. Albendazole efficacy ranged from −35.48% to 62.5%, ivermectin from −2.41% to 51.47%, and combined treatment from −25% to 48.36%. Negative FECR results reflect an increase in FEC after treatment, indicating treatment failure. These results confirm the resistance to albendazole and ivermectin (FECR < 95%) across all six farms. In the untreated control group, mean EPG values increased from day 0 to day 14 on all farms, reflecting natural fluctuations in egg counts during the study period. Figure 2 illustrates the mean EPG values before and after treatment, along with the FECR results for each anthelmintic agent across six meat goat farms.

**Table 3:** FECR results for each anthelmintic agent before (day 0) and after (day 14) treatment in six meat goat farms.

Farm	Anthelmintic	Mean EPG		FECR%	95% CL (LCL-UCL)	Anthelmintic efficacy

		Day 0	Day 14			
1	Albendazole	1625.00	762.50	53.08	24.26–70.93	Resistant
	Ivermectin	943.75	506.25	46.36	−95.00–85.24	Resistant
	Albendazole and Ivermectin	706.25	693.75	1.77	−150.58–61.49	Resistant
	Control	968.75	2231.25	-	-	-
2	Albendazole	793.75	443.75	44.09	−53.89–79.69	Resistant
	Ivermectin	1356.25	743.75	45.16	−34.58–77.65	Resistant
	Albendazole and Ivermectin	762.50	393.75	48.36	−11.09–75.99	Resistant
	Control	925.00	943.75	-	-	-
3	Albendazole	2556.25	2043.75	20.05	−58.70–59.72	Resistant
	Ivermectin	1631.25	1525.00	6.51	−62.44–46.20	Resistant
	Albendazole and Ivermectin	2012.50	1256.25	37.58	−27.04–69.33	Resistant
	Control	1237.50	1400.00	-	-	-
4	Albendazole	2518.75	3412.50	−35.48	−198.97–38.60	Resistant
	Ivermectin	5593.75	4068.75	27.26	−38.95–61.92	Resistant
	Albendazole and Ivermectin	2512.5	2918.75	−16.17	−109.47–35.57	Resistant
	Control	2737.50	2987.50	-	-	-
5	Albendazole	900.00	337.50	62.50	31.05–79.61	Resistant
	Ivermectin	850.00	412.50	51.47	−28.12–81.62	Resistant
	Albendazole and Ivermectin	1375.00	800.00	41.82	−43.60–76.43	Resistant
	Control	837.50	875.00	-	-	-
6	Albendazole	2237.50	1812.50	18.99	−33.42–50.82	Resistant
	Ivermectin	2331.25	2387.50	−2.41	−83.48–42.84	Resistant
	Albendazole and Ivermectin	4400.00	5500.00	−25	−139.13–34.66	Resistant
	Control	2525.00	4143.75	-	-	-

FECR=Fecal egg count reduction, EPG=Eggs per gram, CL=Confidence level, LCL=Lower confidence level, UCL=Upper confidence level

### Strongyle L3 identification by microscopy and semi-nested PCR

All six farms harbored *Haemonchus*, *Trichos-trongylus*, and *Oesophagostomum* before treatment (day 0), with *Haemonchus* being the most prevalent, followed by *Trichostrongylus*; *Oesophagostomum* appeared in low numbers. After treatment (day 14), only *Haemonchus* and *Trichostrongylus* were detected, with *Haemonchus* still dominating the parasite population. The detection of strongyle genus by treatment and farm is presented in Table 4. All morphologically identified *Haemonchus*, *Trichostrongylus*, and *Oesophagostomum* L3 were confirmed by semi-nested PCR.

**Table 4:** Identification of strongyle genera based on microscopic examination of L3 before and after anthelmintic treatment in six meat goat farms.

Farm	Anthelmintic	L3 number (before/after treatment)			

		*Haemonchus*	*Trichostrongylus*	*Oesophagostomum*	Total
1	Control	65/80	32/18	3/2	100/100
	Albendazole	40/46	60/38	0/0	100/84
	Ivermectin	72/36	28/13	0/0	100/49
	Albendazole and Ivermectin	80/93	20/7	0/0	100/100
2	Control	66/2	34/10	0/0	100/12
	Albendazole	62/38	36/62	2/0	100/100
	Ivermectin	28/49	72/51	0/0	100/100
	Albendazole and Ivermectin	3/17	9/75	0/0	12/92
3	Control	35/58	53/32	12/10	100/100
	Albendazole	70/77	20/23	10/0	100/100
	Ivermectin	60/83	36/17	4/0	100/100
	Albendazole and Ivermectin	45/57	39/43	16/0	100/100
4	Control	100/10	0/1	0/0	100/11
	Albendazole	90/88	10/12	0/0	100/100
	Ivermectin	95/96	5/4	0/0	100/100
	Albendazole and Ivermectin	86/92	14/8	0/0	100/100
5	Control	10/6	88/28	2/29	100/63
	Albendazole	8/22	87/42	5/0	100/64
	Ivermectin	12/37	69/4	19/0	100/41
	Albendazole and Ivermectin	4/38	96/62	0/0	100/100
6	Control	80/27	11/31	9/42	100/100
	Albendazole	39/30	18/20	43/0	100/50
	Ivermectin	17/38	57/17	10/0	84/55
	Albendazole and Ivermectin	47/63	31/37	22/0	100/100

L3=Third-stage larvae

### Detection of BZ resistance in strongyle L3 using AS-PCR

**Figure 3:**
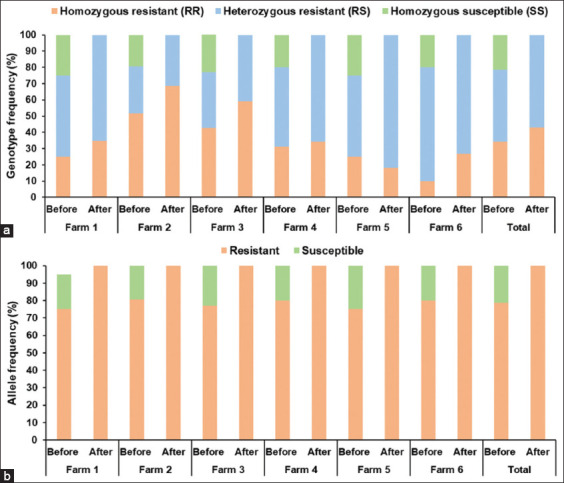
Distribution of (a) genotypic and (b) allelic frequencies associated with benzimidazole resistance in *Haemonchus contortus* L3 before and after albendazole treatment across six meat goat farms.

**Figure 4:**
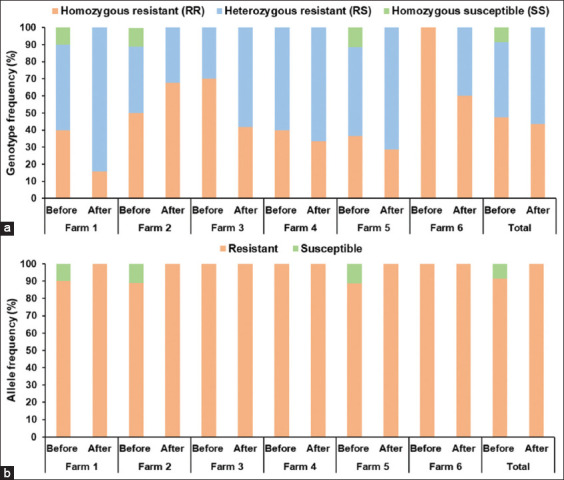
Distribution of (a) genotypic and (b) allelic frequencies associated with benzimidazole resistance in *Trichostrongylus colubriformis* L3 before and after albendazole treatment across six meat goat farms.

The AS-PCR was used to detect the F200Y mutation in the β-tubulin isotype 1 gene associated with BZ resistance in *H. contortus* and *T. colubriformis* L3 from all six farms. The genotypic and allelic frequencies of *H. contortus* before and after treatment are shown in Table 5 and Figure 3, whereas the data for *T. colubriformis* are presented in Table 6 and Figure 4. All three genotypes (RR, RS, and SS) were detected before treatment, but only the RR and RS genotypes remained after treatment. In *H. contortus* L3 (Table 5 and Figure 3), the pretreatment genotype frequencies were RR (34.2%), RS (44.5%), and SS (21.3%). Post-treatment, only the RR (43.0%) and RS (57.0%) genotypes were observed. The resistance allele frequency in *H. contortus* was 78.7% before and 100% after treatment; the susceptible allele (21.3%) was eliminated after treatment.

**Table 5:** Genotypic and allelic frequencies of BZ resistance in *H. contortus* L3 across six meat goat farms, determined using AS-PCR.

Farm	Before treatment				After treatment			% Allele frequency (before/after treatment)	
	
	Number of larvae	Genotype frequency (%)			Number of larvae	Genotype frequency (%)			
		
		RR	RS	SS		RR	RS	Resistant (R)	Susceptible (S)
1	20	5 (25.0)	10 (50.0)	5 (25.0)	23	8 (34.8)	15 (65.2)	75.0/100.0	25.0/0.0
2	31	16 (51.6)	9 (29.0)	6 (19.4)	19	13 (68.4)	6 (31.6)	80.6/100.0	19.4/0.0
3	35	15 (42.8)	12 (34.3)	8 (22.9)	39	23 (59.0)	16 (41.0)	77.1/100.0	22.9/0.0
4	45	14 (31.1)	22 (48.9)	9 (20.0)	44	15 (34.1)	29 (65.9)	80.0/100.0	20.0/0.0
5	4	1 (25.0)	2 (50.0)	1 (25.0)	11	2 (18.2)	9 (81.8)	75.0/100.0	25.0/0.0
6	20	2 (10.0)	14 (70.0)	4 (20.0)	15	4 (26.7)	11 (73.3)	80.0/100.0	20.0/0.0
Total	155	53 (34.2)	69 (44.5)	33 (21.3)	151	65 (43.0)	86 (57.0)	78.7/100.0	21.3/0.0

BZ=Benzimidazole, *H. contortus*=*Haemonchus contortus*, L3=Third-stage larvae, AS-PCR=Allele-specific polymerase chain reaction, RR=Homozygous resistant genotype, RS=Heterozygous resistant genotype, SS=Homozygous susceptible genotype

**Table 6:** Genotypic and allelic frequencies of BZ resistance in *T. colubriformis* L3 across six meat goat farms, determined using AS-PCR.

Farm	Before treatment				After treatment			% Allele frequency (before/after treatment)	
	
	Number of larvae	Genotype frequency (%)			Number of larvae	Genotype frequency (%)	
		
		RR	RS	SS		RR	RS	Resistant (R)	Susceptible (S)
1	30	12 (40.0)	15 (50.0)	3 (10.0)	19	3 (15.8)	16 (84.2)	90.0/100.0	10.0/0.0
2	18	9 (50.0)	7 (38.9)	2 (11.1)	31	21 (67.7)	10 (32.3)	88.9/100.0	11.1/0.0
3	10	7 (70.0)	3 (30.0)	0 (0.0)	12	5 (41.7)	7 (58.3)	100.0/100.0	0.0/0.0
4	5	2 (40.0)	3 (60.0)	0 (0.0)	6	2 (33.3)	4 (66.7)	100.0/100.0	0.0/0.0
5	44	16 (36.4)	23 (52.2)	5 (11.4)	21	6 (28.6)	15 (71.4)	88.6/100.0	11.4/0.0
6	9	9 (100.0)	0 (0.0)	0 (0.0)	10	6 (60.0)	4 (40.0)	100.0/100.0	0.0/0.0
Total	116	55 (47.4)	51 (44.0)	10 (8.6)	99	43 (43.4)	56 (56.6)	91.4/100.0	8.6/0.0

BZ=Benzimidazole, *T. colubriformis=Trichostrongylus colubriformis*, L3=Third-stage larvae, AS-PCR=Allele-specific polymerase chain reaction, RR=Homozygous resistant genotype, RS=Heterozygous resistant genotype, SS=Homozygous susceptible genotype

In *T. colubriformis* L3 (Table 6 and Figure 4), the pretreatment genotypes were RR (47.4%), RS (44.0%), and SS (8.6%). After treatment, only the RR (43.4%) and RS (56.6%) genotypes were detected. The resistance allele frequency in *T. colubriformis* was 91.4% before treatment and reached 100% after treatment; the 8.6% susceptible allele was eliminated.

## DISCUSSION

### First report of AR and molecular surveillance in Nakhon Si Thammarat

To the best of our knowledge, this is the first study in Nakhon Si Thammarat to evaluate AR in GINs for albendazole and ivermectin and detect BZ resistance in *H. contortus* and *T. colubriformis* L3 through F200Y mutation screening in the β-tubulin isotype 1 gene.

### Widespread resistance to common anthelmintics

AR to albendazole, ivermectin, and their combination was observed in all six farms. These findings are consistent with reports from other Thai provinces, Narathiwat, Ratchaburi, Sing Buri, Chaiyaphum, and Khon Kaen [22–25], and from countries such as Sudan, India, Ethiopia, and the Czech Republic [8, 9, 17, 33]. The extensive and long-term use of albendazole and ivermectin in the region likely contributed to the observed widespread resistance. In addition, farmers frequently depend on visual weight estimation to calculate anthelmintic doses, which can lead to underdosing, a significant contributor to AR development, or overdosing that unnecessarily increases treatment costs [34]. Although farmers practiced rotation between albendazole and ivermectin, it was likely introduced after resistance had already emerged, rendering the strategy ineffective. The combination therapy exhibited limited efficacy in eradicating GINs, potentially attributable to cross-resistance between BZs and MLs, as evidenced by the identical nucleotide alterations in the β-tubulin isotype 1 gene found in both ivermectin-resistant *H. contortus* and BZ-resistant strains [35]. The introduction of alternative anthelmintic classes with different modes of action from those of existing anthelmintics is recommended for routine deworming and as a preventative strategy before selling live goats to other farms. Such measures may enhance parasite control and help prevent further AR dissemination. In Thailand, the availability of effective alternative anthelmintics, such as levamisole, is currently limited and may require further efficacy trials before widespread application [22–24].

### Post-treatment nematode persistence confirms resistance

The persistence of *Haemonchus* and *Trichos-trongylus* L3 after treatment with albendazole, ivermectin, and their combination confirms their resistance to these drugs. The absence of *S. papillosus* and *Trichuris* eggs and *Oesophagostomum* L3 after treatment suggests continued susceptibility of these species to the administered drugs. Similar results were reported in Ratchaburi, Thailand, where *Haemonchus* and *Trichostrongylus* were detected after treatment with albendazole and ivermectin [24]. *Haemonchus* and *Trichostrongylus* persistence after albendazole or ivermectin treatment has also been documented in Sudan, India, Ethiopia, and the Czech Republic [8, 9, 17, 33].

### High prevalence of the F200Y mutation confirms BZ resistance

This study detected a high frequency of the F200Y mutation linked to BZ resistance in the β-tubulin isotype 1 gene of *H. contortus* and *T. colubriformis* in Nakhon Si Thammarat goats. These molecular findings are supported by FECR results showing low albendazole efficacy (−35.48%–62.5%) in the region. In *H. contortus*, the frequencies of resistant alleles ranged from 75.0% to 80.6% before treatment and increased to 100% afterward, suggesting the elimination of susceptible genotypes. The prevalence of resistant alleles in *H. contortus* reported here exceeds that reported by Pitaksakulrat *et al*. [26], who observed frequencies of 30%–65% across different Thai regions. The prevalence of resistant alleles varies globally, with reported rates of 60.5%–88% in India [17, 36], 35.7% in Mozambique [19], 2%–31% in China [37], 86% in Malaysia [38], 100% in Greece [39], 27.5%–52.5% in Bangladesh [40], 10.5%–74% in Slovakia [41], and 100% in Bosnia and Herzegovina [42]. Few studies have reported F200Y-associated BZ resistance in *T. colubriformis*, and most of these studies have focused on sheep rather than goats. In this study, the prevalence of BZ-resistant alleles in *T. colubriformis* ranged from 88.6% to 100% before treatment and reached 100% after treatment, indicating the loss of susceptible genotypes. These results show a higher prevalence of BZ-resistant alleles in goats from India (6.7%–95%) [43] and sheep from Spain (89.8%–99.3%) [44], Malaysia (87%) [38], and Austria (77%–100%) [20]. AS-PCR remains a useful tool for tracking RS and RR genotypes in nematode populations, allowing early detection of BZ resistance. The predominance of RS genotypes over RR genotypes in both species emphasizes the need for timely interventions to prevent progression toward complete RR dominance. The recommended strategies include combination therapy, anthelmintic class rotation, targeted selective treatment, pasture rotation, mixed-species grazing, and routine resistance monitoring.

### Integration of FECR and molecular methods enhances surveillance

The FECR test is a phenotypic method commonly used to evaluate anthelmintic efficacy across all classes; however, it could lead to an underestimation of BZ resistance on farms where the resistant nematode population was below 25% [45]. Molecular approaches, such as AS-PCR, improve genotypic diagnosis, allowing for the early and specific detection of BZ-resistant alleles. Consequently, the integration of both approaches enhances the accuracy and sensitivity of resistance surveillance, thereby supporting more effective parasite management strategies.

### Impact of AR on goat farming and public health

AR emergence in *H. contortus* and *T. colubriformis*, the two most harmful nematodes, poses a significant threat to the health and economic sustainability of goat farming. AR reduces the effectiveness of helminth control, leading to decreased productivity and increased morbidity, thereby lowering farm income and increasing veterinary expenses [46]. In addition, these two nematodes, known as zoonotic helminths, pose a significant threat to public health by causing human trichostrongylosis [12–14]. Ineffective control of parasites can result in increased environmental contamination with infective larvae, thereby raising human health risks.

## CONCLUSION

This study provides the first integrated field-based and molecular evaluation of AR in GINs of meat goats in Nakhon Si Thammarat, Southern Thailand. Resistance to albendazole, ivermectin, and their combination was confirmed across all six investigated farms, with FECR values consistently below the 95% efficacy threshold. Albendazole efficacy ranged from −35.48% to 62.5%, ivermectin from −2.41% to 51.47%, and the combination therapy from −25% to 48.36%. Persistent post- treatment detection of *Haemonchus* and *Trichostrongylus* larvae further confirmed treatment failure, while the absence of *Oesophagostomum* indicated continued susceptibility to the administered drugs.

Molecular analysis revealed high frequencies of the F200Y mutation in the β-tubulin isotype 1 gene, a key marker of BZ resistance. Resistant allele frequencies in *H. contortus* ranged from 75.0% to 80.6% before treatment and reached 100% post-treatment. Similarly, *T. colubriformis* showed resistance allele frequencies of 88.6%–100% pre-treatment, increasing to 100% post-treatment. The elimination of susceptible genotypes indicates fixation of resistance in local parasite populations, reflecting trends reported from other endemic regions.

These findings underscore the urgent need for improved parasite control strategies. The long-term and unsupervised use of albendazole and ivermectin, often with visually estimated dosing, has likely accelerated the emergence and spread of AR. Practical interventions such as the use of alternative anthelmintic classes, rotational deworming protocols, targeted selective treatment, and pasture management are essential. Equally important is the need for farmer education and regular monitoring of drug efficacy through both phenotypic and genotypic methods.

The study’s strength lies in its dual-assessment approach combining FECR and AS-PCR, and in its focus on two highly pathogenic and zoonotic GIN species. However, the study was limited by partial molecular screening (50% of larvae) and its focus on a single resistance mutation (F200Y), without examining other potential markers or resistance to MLs and imidazothiazoles.

Future research should aim to explore additional resistance mutations, evaluate the efficacy of underutilized or new drug classes, and conduct longitudinal surveillance of resistance dynamics. Nationwide implementation of molecular diagnostics would provide a more accurate resistance map, enabling evidence-based policymaking for parasite control.

In conclusion, the confirmed MDR in *H. contortus* and *T. colubriformis* represents a serious threat to goat health, productivity, and public health in southern Thailand. Immediate action is needed to integrate sustainable control practices, ensure responsible drug use, and curb the further spread of resistance within and beyond the region.

## AUTHORS’ CONTRIBUTIONS

NS, RM, and MK: Conceptualization and methodology. RM and MK: Supervised the study. NS, CS, and PF: Investigation. NS, CS, and TK: Data curation, validation, formal analysis, software, and visualization. NS: Project administration and writing – original draft preparation. NS, CS, PF, TK, RM, and MK: Reviewed and edited the manuscript. All authors have read and approved the final manuscript.
